# Trends in smoking behaviour among Estonian physicians in 1982–2014

**DOI:** 10.1186/s12889-017-4596-x

**Published:** 2017-07-25

**Authors:** Kersti Pärna, Mariliis Põld, Inge Ringmets

**Affiliations:** 1 Institute of Family Medicine and Public Health, University of Tartu, Ravila 19, 50411 Tartu, Estonia; 2 Estonian Health Insurance Fund, Lastekodu 48, 10144 Tallinn, Estonia

**Keywords:** Smoking, Physicians, Trends, Estonia

## Abstract

**Background:**

Smoking surveys among physicians have proved useful in highlighting the importance of physicians as healthy life style exemplars and role models in tobacco control and smoking cessation. The aim of this study was to give an overview of smoking behaviour among Estonian physicians from 1982 to 2014.

**Methods:**

Three cross-sectional postal surveys using a self-administered questionnaire were carried out among all practising physicians in Estonia. The number of physicians participating in this study was 3786 in 1982, 2735 in 2002, and 2902 in 2014. Data analysis involved calculating the age-standardized prevalences of smoking, prevalences of smoking by age group and mean age of smoking initiation. A non-parametric test for trend was used to assess significant changes in smoking over time.

**Results:**

Age-standardized prevalence of current smoking among men was 39.7% in 1982, 20.9% in 2002, and 14.3% in 2014 and among women 12.2%, 8.0%, and 5.2%, respectively (*p* < 0.0001 for trends). From 1982 to 2014, the biggest decline of current smoking among men and women was in age groups under 35 (from 55.2% to 16.7% and from 16.7% to 2.8%, respectively) and 35–44 (from 47.1% to 8.3% and from 19.5% to 5.1%, respectively) (*p* < 0.0001 for trends). Mean age of smoking initiation decreased from 20.4 to 19.3 among men and from 24.5 to 20.4 among women over the study period.

**Conclusions:**

In 1982–2014, smoking prevalence among Estonian physicians declined substantially. This may influence the willingness of society to recognize the health consequences of smoking which could give a support to the decline of the smoking epidemic in the country. Differences between smoking among male and female physicians persisted over the study period, but mean age of smoking initiation decreased. A further decline in smoking among Estonian physicians should be encouraged by special efforts targeted at physicians.

## Background

The tobacco epidemic is one of the biggest preventable public health threats, killing around 6 million people a year [1]. Smoking cessation counselling by physicians has been effective in increasing cessation rates, but their own smoking habits are known to influence their effectiveness in this role [2, 3]. Physicians are often viewed as healthy lifestyle models by their patients and the communities in which they live [2]. Early age of initiating smoking has an important role in becoming a smoker [4]. Moreover, the prevalence of smoking among physicians may reflect the ‘maturity’ of the smoking epidemic in a particular country. When the prevalence of smoking among physicians falls below that of general population, the country’s smoking epidemic can be considered to be ‘mature’ [5].

Tobacco smoking by physicians in different countries has been the subject of epidemiological studies for a long time [6–9]. These studies have shown that smoking prevalence among physicians in high income countries such as Great Britain, the United States, Australia and Scandinavian countries, has decreased during the last decades. Also, smoking prevalence among the total population in developed countries has decreased during the last decades [10].

In Estonia, data concerning smoking among 16–64-years-olds has been available since 1990 and shows a decreasing trend. While in 1990 the prevalence of daily smoking was 48% among men and 15% among women [11], by 2014 the rates were 31% and 15%, respectively [12]. In 1990–2014, the highest prevalence of smoking was in 1994 when 50% of men and 21% of women were daily smokers [13]. In Estonia, surveys concerning smoking among physicians have been carried out four times (1978, 1982, 2002, 2014), but trends in smoking habits among Estonian physicians during the last three decades have not been reported.

The aim of this study was to analyse trends in smoking behaviour among Estonian physicians from 1982 to 2014.

## Methods

### Study population and design

The present study was based on three smoking surveys among Estonian physicians in 1982, 2002, and 2014. Unfortunately the data of the first survey in 1978 were not available.

The methods used in the surveys in 1982, 2002 and 2014 have been extensively described elsewhere [14–19]. In short, all practicing physicians who were permanent residents of Estonia were eligible for sampling in all study years. Cross-sectional postal surveys using similar, self-administered questionnaires were used to collect information on smoking in 1982, 2002, and 2014. In 1982, all practicing physicians were identified from the database of the Ministry of Health in Estonia. In 2002, all practicing physicians were identified from the database of Estonian Health Insurance Fund. In these two study years questionnaires were mailed to the workplace address of the physicians. In 2014, the sample of all practicing physicians was based on data from the Estonian Health Care Professionals Registry and questionnaires were mailed to the home address of physicians. To receive home addresses, data from the Estonian Health Care Professionals Registry were linked with the Population Registry in Estonia.

The number of respondents was 3792 in 1982, 2747 in 2002, and 2903 in 2014 (Table 1). The crude response rates were 80.7%, 66.3%, and 52.0%, respectively. The corrected response rates (excluding the physicians who were unavailable, retired, had an incorrect address, had left Estonia or died) were available only for the last two study years, being 67.8% in 2002 and 53.1% in 2014.

**Table 1:** Initial sample size, number of respondents, crude and corrected response rates among physicians by gender and study year in Estonia

Study year	Initial sample size	Number of respondents			Response rate (%)	
		Men	Women	Total	Crude	Corrected
1982	4704	901	2891	3792	80.7	-
2002	4140	471	2276	2747	66.3	67.8
2014	5666	532	2371	2903	52.0	53.1

### Study variables

Age was measured in full years and analyzed in five groups: −34, 35–44, 45–54, 55–64, 65 + .

Age of initiation of smoking among current smokers was measured in full years.

Smoking status was determined by combining answers to several questions concerning daily, occasional, past and never smoking and classified as current (daily and occasional) smoking and non-smoking (past and never smoking).

### Statistical analysis

Data analysis involved the determination of prevalence estimates and the corresponding 95% confidence intervals (CI). Calculation of age-standardized prevalence of smoking using European standard population [20] was performed. Mean age of respondents with standard deviation (SD), minimum (min) and maximum (max) value, and mean age of smoking initiation with 95% CI were calculated. A non-parametric test for trend was used to assess significant changes in smoking over time [21].

In 1982 six physicians, in 2002 twelve, and in 2014 one physician did not answer the questions concerning smoking and were excluded from the analysis. A total of 3786 questionnaires from 1982, 2735 from 2002, and 2902 from 2014 were used in the analysis. Questionnaires with missing information concerning age of smoking initiation (1.8% of questionnaires over the study period) were excluded from the calculation of mean age of smoking initiation.

Data were analyzed using statistical package Stata 12 [22].

## Results

### Mean age of physicians

The mean age of men was 44.8 (SD 12.3, min 23, max 79) and of women 42.6 years (SD 10.1, min 23, max 79) in 1982, 47.9 (SD 12.1, min 25, max 82) and 47.7 (SD 11.2, min 24, max 84) in 2002, respectively, and 52.7 (SD 14.5, min 25, max 85) and 51.2 (SD 14.0, min 24, max 86) in 2014, respectively.

### Mean age of smoking initation

The mean age of smoking initiation among currently smoking men was 20.4 in 1982, 19.9 in 2002, and 19.3 in 2014 (Table 2). The mean age of smoking initiation among currently smoking women was 24.5 in 1982, 22.3 in 2002, and 20.4 in 2014. From 1982 to 2014, the mean age of onset of smoking decreased by 1.1 years among male and 4.1 years among female physicians.

**Table 2:** The mean age and 95% confidence interval for smoking initiation among current smokers by gender and study year

Study year	Men		Women	
	Mean age	95% CI	Mean age	95% CI
1982	20.4	20.0–20.8	24.5	24.0–25.0
2002	19.9	18.9–20.8	22.3	21.5–23.0
2014	19.3	18.1–20.5	20.4	19.7–21.1

### Age-standardized prevalences of smoking

Age-standardized prevalence of current smoking among physicians was 19.0% (95% CI 17.0–21.0) in 1982, 12.7% (95% CI 9.3–11.5) in 2002 and 7.0% (95% CI 5.8–8.1) in 2014.

**Fig. 1:**
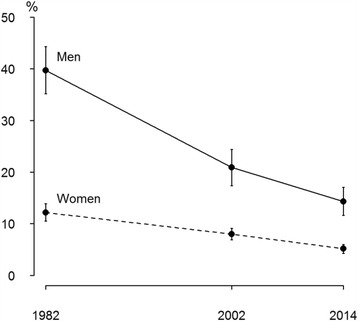
Age-standardized prevalence of current smoking (%) over time by gender among physicians in Estonia, 1982–2014

Age-standardized prevalence of current smoking among men was 39.7% (95% CI 35.2–44.3) in 1982, 20.9% (95% CI 17.4–24.4) in 2002 and 14.3% (95% CI 11.6–17.1) in 2014, among women 12.2% (95% CI 10.5–13.9), 8.0% (95% CI 6.9–9.1) and 5.2% (95% CI 4.3–6.0), respectively (Fig. 1). Overall, age-standardized prevalence of current smoking decreased by 2.8-fold among male and 2.4-fold among female physicians between 1982 and 2014 (*p* < 0.0001 for trends). Compared to women, age-standardized prevalence of current smoking among men was 3.3 times higher in 1982, but 2.8 times higher in 2014.

Age-standardized prevalence of daily smoking was 38.3% (95% CI 33.8–42.9) in 1982, 15.0% (95% CI 11.9–18.1) in 2002 and 11.7% (95% CI 9.2–14.2) in 2014 among men and 10.3% (95% CI 8.9–11.7), 4.7% (95% CI 3.9–5.6) and 3.9% (95% CI 3.2–4.7), respectively, among women. Overall, age-standardized prevalence of daily smoking decreased by 3.3-fold among male and 2.6-fold among female physicians between 1982 and 2014. Compared to women, age-standardized prevalence of daily smoking was 3.7 times higher among men in 1982, but 3.0 times higher in 2014.

Age-standardized prevalence of occasional smoking was 1.4 (95% CI 0.7–2.0) in 1982, 5.9% (95% CI 3.8–8.0) in 2002 and 2.6% (95% CI 1.3–4.0) in 2014 among men, 1.9% (95% CI 0.9–2.9), 3.3% (95% CI 2.5–4.0) and 1.2 (95% CI 0.8–1.7), respectively, among women.

Age-standardized prevalence of past smoking among men increased from 24.2% (95% CI 20.2–28.2) in 1982 to 28.3% (95% CI 24.3–32.4) in 2002, and thereafter decreased to 25.0% 95% CI 21.9–28.2) in 2014. Among women the age-standardized prevalence of past smoking increased from 10.6% (95% CI 8.4–12.8) in 1982 to 16.3% (95% CI 14.6–18.0) in 2014. Compared to women, age-standardized prevalence of past smoking among men was 2.3 times higher in 1982, but 1.5 times higher in 2014.

In 1982–2014, age-standardized prevalence of physicians who had never smoked increased from 33.3% (95% CI 29.1–37.5) to 50.8% (95% CI 47.1–54.5) among men and from 74.4% (95% CI 71.8–77.1) to 78.5% (95% CI 76.7–80.3) among women. Compared to women, age-standardized prevalence of never smoking among men was 2.2 times lower in 1982, but 1.5 times lower in 2014.

### Smoking by age groups

**Fig. 2:**
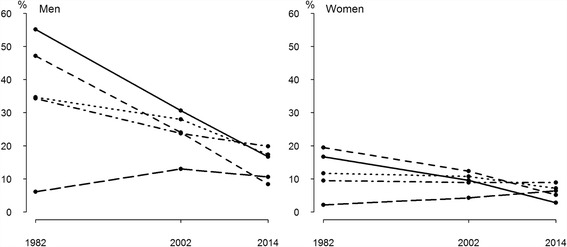
Prevalence of current smoking (%) over time by gender and age group among physicians in Estonia, 1982–2014

In 1982–2014, the biggest decrease of current smoking among men and women was in those aged −34 (55.2% and 16.7% for men, 16.7% and 2.8% for women, repectively) and in age group 35–44 (47.1% and 8.3% for men, 19.5% and 5.1% for women, respectively) (*p* < 0.0001 for trends) (Fig. 2). Current smoking decreased slightly but significantly among 45–54- and 55–64-year-old men (*p* = 0.0006 and *p* = 0.0064, respectively), but increased slightly among men in the oldest age group (65+) over the study period (*p* = 0.4289). While in 1982 and 2002 the highest prevalence of current smoking among men was in those aged −34 (55.2% and 30.7%, respectively) followed by those aged 35–44 (47.1%) in 1982 and 45–54 (28.0%) in 2002, then in 2014 in those aged 55–64 (19.8%), followed by those aged 45–54 (17.3%) and −34 (16.7%).

Among women, current smoking decreased in the 45–54 age group (*p* = 0.0089), was stable among 55–64-year-olds (*p* = 0.7509), but increased slightly in the oldest age group (65+) (*p* = 0.1563) (Fig. 2). While in 1982 and 2002 the highest prevalence of current smoking among women was in the age group 35–44 (19.5% and 12.3%, respectively) followed by the age group −34 (16.7%) in 1982 and 45–54 (10.7%) in 2002, then in 2014 in age group 55–64 (8.9%), followed by age groups 45–54 (7.1%) and 65+ (6.4%).

While daily smoking prevalence among men was the highest in the youngest age group in 1982 (53.4%), by 2002 it was the highest among 45–54-year-olds (20.8%) and in 2014 among 55–64-year-olds (15.1%) (Table 3). Daily smoking among women was the highest among those aged 35–44 in 1982 (17.0%) and 2002 (7.2%), but among 55–64-year-olds in 2014 (8.0%). Daily smoking was the lowest among men and women in the oldest age group in all study years. In 2014, the lowest daily smoking prevalence was among 35–44-year-old men and in the youngest age group of women.

**Table 3:** Smoking status among Estonian male and female physicians by age group and study year (n, %, 95% CI), 1982–2014

Smoking status	Age groups					
	−34	35–44	45–54	55–64	65+	Total
1982						
Men						
	*n* = 232	*n* = 227	*n* = 251	*n* = 140	*n* = 49	*n* = 899
Daily	53.4 (46.8–60.0)	44.9 (38.3–51.7)	33.1 (27.3–39.3)	32.1 (24.5–40.6)	6.1 (1.3–16.9)	39.7 (36.5–43.0)
Occasional	1.7 (0.5–4.4)	2.2 (0.7–5.1)	1.6 (0.4–4.0)	2.1 (0.4–6.1)	0.0 (0.0–7.3)^a^	1.8 (1.0–2.9)
Past	13.4 (9.3–18.4)	17.6 (12.9–23.2)	30.3 (24.7–36.4)	35.7 (27.8–44.2)	40.8 (27.0–55.8)	24.1 (21.4–27.1)
Never	31.5 (25.5–37.9)	35.2 (29.0–41.8)	35.1 (29.2–41.3)	30.0 (22.6–38.3)	53.1 (38.3–67.5)	34.4 (31.3–37.6)
Women						
	*n* = 737	*n* = 970	*n* = 796	*n* = 337	*n* = 47	*n* = 2887
Daily	13.8 (11.4–16.5)	17.0 (14.7–19.5)	10.4 (8.4–12.8)	9.2 (6.3–12.8)	2.1 (0.1–11.3)	13.2 (12.0–14.5)
Occasional	2.8 (1.8–4.3)	2.5 (1.6–3.7)	1.3 (0.6–2.3)	0.3 (0.0–1.6)	0.0 (0.0–7.5)^a^	1.9 (1.5–2.5)
Past	11.4 (9.2–13.9)	9.6 (7.8–11.6)	7.4 (5.7–9.5)	8.9 (6.1–12.5)	19.1 (9.1–33.3)	9.5 (8.5–10.7)
Never	71.9 (68.5–75.1)	70.9 (68.0–73.8)	80.9 (78.0–83.6)	81.6 (77.0–85.6)	78.7 (64.3–89.3)	75.3 (73.7–76.9)
2002						
Men						
	*n* = 62	*n* = 150	*n* = 125	*n* = 80	*n* = 54	*n* = 471
Daily	17.7 (9.2–29.5)	17.3 (11.6–24.4)	20.8 (14.1–29.0)	17.5 (9.9–27.6)	11.1 (4.2–22.6)	17.6 (14.3–21.4)
Occasional	12.9 (5.7–23.9)	6.7 (3.2–11.9)	7.2 (3.3–13.2)	6.3 (2.1–14.0)	1.9 (0.0–9.9)	7.0 (4.9–9.7)
Past	19.4 (10.4–31.4)	33.3 (25.9–41.5)	33.6 (25.4–42.6)	33.8 (23.6–45.2)	40.7 (27.6–55.0)	32.5 (28.3–36.9)
Never	50.0 (37.0–63.0)	42.7 (34.6–51.0)	38.4 (29.8–47.5)	42.5 (31.5–54.1)	46.3 (32.6–60.4)	42.9 (38.4–47.5)
Women						
	*n* = 305	*n* = 657	*n* = 644	*n* = 516	*n* = 142	*n* = 2264
Daily	5.2 (3.0–8.4)	7.2 (5.3–9.4)	6.5 (4.7–8.7)	5.8 (4.0–8.2)	2.8 (0.8–7.1)	6.1 (5.2–7.2)
Occasional	4.3 (2.3–7.2)	5.2 (3.6–7.2)	4.2 (2.8–6.0)	3.1 (1.8–5.0)	1.4 (0.2–5.0)	4.1 (3.3–5.0)
Past	12.5 (9.0–16.7)	16.0 (13.3–19.0)	18.3 (15.4–21.5)	18.2 (15.0–21.8)	10.6 (6.0–16.8)	16.3 (14.8–17.9)
Never	78.0 (73.0–82.6)	71.7 (68.1–75.1)	71.0 (67.3–74.4)	72.9 (68.8–76.7)	85.2 (78.3–90.6)	73.5 (71.6–75.3)
2014						
Men						
	*n* = 72	*n* = 84	*n* = 127	*n* = 126	*n* = 123	*n* = 532
Daily	13.9 (6.9–24.1)	4.8 (1.3–11.7)	14.2 (8.6–21.5)	15.1 (9.3–22.5)	10.6 (5.7–17.4)	12.0 (9.4–15.1)
Occasional	2.8 (0.3–9.7)	3.6 (0.7–10.1)	3.1 (0.9–7.9)	4.8 (1.8–10.1)	0 (0–3.0)^a^	2.8 (1.6–4.6)
Past	4.2 (0.9–11.7)	23.8 (15.2–34.3)	40.2 (31.6–49.2)	38.9 (30.3–48.0)	39.8 (31.1–49.1)	32.3 (28.4–36.5)
Never	79.2 (68.0–87.8)	67.9 (56.8–77.6)	42.5 (33.8–51.6)	41.3 (32.6–50.4)	49.6 (40.5–58.8)	52.8 (48.5–57.1)
Women						
	*n* = 395	*n* = 353	*n* = 606	*n* = 575	*n* = 441	*n* = 2370
Daily	1.8 (0.7–3.6)	3.4 (1.8–5.9)	5.1 (3.5–7.2)	8.0 (5.9–10.5)	5.4 (3.5–8.0)	5.1 (4.2–6.0)
Occasional	1.0 (0.3–2.6)	1.7 (0.6–3.7)	2.0 (1.0–3.4)	0.9 (0.3–2.0)	0.9 (0.2–2.3)	1.3 (0.9–1.9)
Past	11.6 (8.7–15.2)	10.8 (7.8–14.5)	19.5 (16.4–22.9)	26.3 (22.7–30.1)	22.4 (18.6–26.6)	19.1 (17.5–20.7)
Never	85.6 (81.7–88.9)	84.1 (79.8–87.8)	73.4 (69.7–76.9)	64.9 (60.8–68.8)	71.2 (66.7–75.4)	74.6 (72.8–76.3)

^a^one-sided 97.5% confidence interval

Prevalence of occasional smoking was less than 5.0% among men and women in all age groups in 1982 and 2014 (Table 3). In 2002, the prevalence of occasional smoking was somewhat higher being greatest among under 35-year-old men (12.9%) and 35–44-year-old women (5.2%). While in 1982 and 2002 the prevalence of past smoking among men was higher in older age groups, then in 2014 the highest prevalence of past smoking was among 45–54-year-olds. Prevalence of physicians who had never smoked was in the youngest age group of men 31.5% in 1982, 50.0% in 2002 and 79.2% in 2014 (among women 71.9%, 78.0% and 85.6%, respectively).

## Discussion

The main research question of this study was to analyse trends in smoking behaviour among Estonian physicians.

Before discussing the results some limitations of this study should be addressed. *First*, response rates in the three physician surveys declined over time, as is the case with postal surveys in most populations worldwide. The non-responders may have revealed different patterns of smoking compared with those who returned the questionnaire. At the same time, the respondents were representative of the overall physician population by men-women ratio in Estonia, with slightly higher proportion of respondents among women. *Second*, self-reports of smoking tends to slightly underestimate the actual amount of smoking among physicians. As physicians know more about the devastating effects of smoking than the general population, they may be especially prone to self-deception or understatement. *Third*, as the data of the first study were collected in the Soviet era, and some minor changes were made in study design and questions between studies, the findings of trends in the study need to be interpreted with caution. Despite these caveats, several inferences can be drawn.

### Trends in smoking

The findings highlight several trends in smoking behaviour in Estonia.

*First*, the age-standardized prevalence of current smoking among physicians declined by nearly three times from 1982 to 2014 in Estonia. In 1982, about one fifth, but in 2014, less than one tenth of physicians were current smokers. Most developed countries (e.g. Great Britain, Australia, United States, Finland, and Japan) appear to have experienced a similar steady decline in smoking prevalence among physicians over the past decades [6–9, 16, 23, 24].

*Second,* despite of overall decline in smoking, the prevalence of current smoking among physicians in Estonia was still higher than in most developed countries. For example, the prevalence of smoking among physicians in Estonia in 2014 was comparable with smoking among physicians in Australia and the United States in the 1980s [7, 25]. However, smoking prevalence in some developed countries (e.g Japan, Italy, and France) have remained high with prevalence of current smoking over 25% [8, 26, 27].

*Third,* the age-standardized prevalence of current smoking in Estonia declined among male as well as among female physicians over the study period. In 1982, more than one third of male and one tenth of female physicians were current smokers. By 2014, only about one seventh of male and one twentieth of female physicians reported themselves to be current smokers. Thus, age-standardized prevalence of current smoking decreased by nearly three-fold among men and more than two-fold among women over the study period. In 1982–2014, the biggest decline in current smoking was among male and female physicians in younger age groups (−34, 35–44) in Estonia. The same trend was seen in age-standardized prevalence of daily smoking among physicians in Estonia. While in 1982 more than one third of male and one tenth of female physicians were daily smokers, by 2014 the age-standardized prevalence was more than three times lower among male and nearly three times lower among female physicians (11.7% and 3.9%, respectively). The age-standardized prevalence of past smoking among men increased 1.2-fold in 1982–2002 and thereafter slightly decreased, but among women increased 1.5-fold over study period. The age-standardized prevalence of never having smoked increased 1.5 times among men, but was stable among women physicians in 1982–2014. At the same time, the biggest increase in the prevalence of never having smoked was among male and female physicians in younger age groups which means that non-smokers entering to the profession are also a source of change in smoking prevalence among physicians. Thus, the decline in smoking among physicians in Estonia was the result of an increase in quitting behaviour and possibly in the increasing number of nonsmokers becoming physicians. Similarly, the decline in smoking among physicians observed in the United States has been the result of an increase of both quitting behaviour and in the number of nonsmokers entering the occupation [25].

*Fourth*, compared to the first study year, Estonian physicians, especially women, started smoking at a younger age in the last study year. It is well established, that early age of starting smoking is one of the determinant factors of nicotine dependence and smoking temptation [4, 30]. One reason why earlier onset did not appear to influence the declining trend in smoking among physicians in Estonia, could be explained by the increasing number of nonsmokers entering the occupation. Based on the results of Estonian Health Interview Surveys in 1996 and 2006, the trend in onset of smoking among adult population seems to be the same among the adult population in Estonia [28, 29]. One explanation of the earlier start of smoking could be the heavy marketing by tobacco companies flooding Estonia after the collapse of the Soviet Union in 1991.

*Fifth,* almost all currently smoking male and female physicians in Estonia were daily smokers as age-standardized prevalence of occasional smoking varied from one to 3 % in 1982 and 2014 being somewhat higher in 2002 (5.9% among men and 3.3% among women). Conversely, only one third of current physician smokers in Finland were daily smokers [16]. The high percentage of daily smoking among current smokers in Estonia could be explained by the decreasing age of onset of smoking over the study period. According to the worldwide literature there is much evidence that those who start smoking earlier are more prone to become heavy smokers than those who start later [4, 30].

*Sixth*, despite the overall decline in smoking, the difference between men and women persisted in Estonia being about three time higher among men than women. Smoking among the general population is similar with much higher smoking prevalences among men in Central and Eastern Europe than in Western European and Scandinavian countries [10]. Smoking among physicians by gender differs by country. In Armenia [31] and Japan [32], smoking is more common among male physicians, while in Italy [33], female physicians smoke at higher rates, but in Australia [6, 7] and the United States [6, 9], smoking prevalence is almost the same between the sexes.

*Seventh,* the prevalence of smoking among physicians was much lower than that observed in the general population and in the highest educational bracket of the total population in Estonia. These findings are consistent with previous studies [16, 17] indicating that physicans are far less likely to smoke than the general population in Estonia. Thus, smoking among physicians in Estonia as in most developed countries [7, 23, 25], is comparable with the „mature **“**smoking epidemic [5]. Nevertheless, in Italy the overall smoking prevalence among Italian physicians was similar to that of the general population in 1995. While male physicians in Italy smoked less frequently than the male general population (25% vs 34%), female physicians smoked more frequently than the female general population (23% vs 17%) [26].

### Tobacco control in Estonia

Tobacco legislation and control in Estonia have become increasingly powerful.

In Estonia, there was no tobacco legislation in the 1980s during the Soviet era and the first years after the collapse of the Soviet Union in 1991, when tobacco policies were based on guidelines from the Ministry of Health. Since 2000, Estonia has witnessed considerable changes in tobacco control. The first Tobacco Act in Estonia was enforced in 2001 [34]. The revised Tobacco Act came into force in June 2005 and brought Estonia in line with the European Union directive on tobacco and the WHO anti-tobacco convention [35]. This law banned smoking completely in catering establishments. In 2014, the Green Paper on Tobacco Policy was published in Estonia to give an overview of necessary measures to be implemented to reduce the prevalence of smoking and tobacco-related health effects [36].

Since 2000, Estonia has belonged to the International Network of Health Promoting Hospitals and Health Services (HPH) [37]. A key strategy of HPHs was creating a smoke free environment in hospitals. In 2015, about 20 hospitals (around 30%) in Estonia joined HPH [38]. In 2005, the Estonian Network of Health Promoting Hospitals and Health Services (Estonian HPH Network) joined the European Network of Smoke-Free Hospitals (ENSH-Global). Since 2015, six leading hospitals in Estonia have become smoke-free [39].

## Conclusion

In conclusion, this study is the first to describe smoking among Estonian physicians over three decades. Overall, it can be clearly seen that from 1982 to 2014 smoking declined among physicians in Estonia. At the same time, physicians started to smoke at an earlier age from 1982 to 2014. The difference in smoking prevalence of male and female physicians persisted over the study period.

It is important that smoking trends among physicians continue to decline so that all physicians can become good role models for the general population. A further decline in smoking among Estonian physicians should be supported by special efforts targeted at physicians.

## References

[1] World Health Organization. Tobacco. Fact sheet. Updated June 2016. http://www.who.int/mediacentre/factsheets/fs339/en/. Accessed 18 Jan 2017.

[2] Garfinkel L (1976). Cigarette smoking among physicians and other health professionals, 1959–1972. CA Cancer J Clin.

[3] Kawakami M, Nakamura S, Fumimoto H, Takizawa J, Baba M (1997). Relation between smoking status of physicians and their enthusiasm to offer smoking cessation advice. Intern Med.

[4] Breslau N, Fenn N, Peterson EL (1993). Early smoking initiation and nicotine dependence in a cohort of young adults. Drug Alcohol Depend.

[5] Davis RM (1993). When doctors smoke. Tob Control.

[6] Smith DR, Leggat PA (2007). An international review of tobacco smoking in the medical profession: 1974–2004. BMC Public Health.

[7] Smith DR, Leggat PA (2008). The historical decline of tobacco smoking among Australian physicians: 1964–1997. Tob Induc Dis.

[8] Smith DR, Wada K (2013). Declining rates of tobacco use in the Japanese medical profession, 1965–2009. J Epidemiol.

[9] Smith DR (2008). The historical decline of tobacco smoking among United States physicians: 1949–1984. Tob Induc Dis.

[10] World Health Organization. European Health for All database (HFA-DB). Updated July 2016. http://www.euro.who.int/en/data-and-evidence/databases/european-health-for-all-database-hfa-db. Accessed 24 Jan 2017.

[11] Lipand A, Kasmel A, Kivilo M (1991). Eesti täiskasvanud elanikkona terviseuurimus 1990.a. kevadel. Health behaviour among Estonian adult population, spring 1990.

[12] Tekkel M, Veideman T. Eesti täiskasvanud rahvastiku tervisekäitumise uuring, 2014. Health behaviour among Estonian adult population, 2014. Tallinn: Tervise Arengu Instituut; 2015.

[13] Lipand A, Kasmel A, Tasa E, et al. Eesti täiskasvanud elanikkonna terviseuuring, kevad 1994. Health behaviour among Estonian adult population, spring 1994. Helsinki: National Public Health Institute; 1995.

[14] Rahu M, Raudsepp J (1986). Teine Eesti NSV arstkonna suitsetamislevimuse ankeetküsitlus 1982. aastal. Nõukogude Eesti Tervishoid.

[15] Innos K, Rahu K, Baburin A, Rahu M (2002). Cancer incidence and cause-specific mortality in male and female physicians: a cohort study in Estonia. Scand J Public Health.

[16] Pärna K, Rahu K, Barengo NC (2005). Comparison of knowledge, attitudes and behaviour regarding smoking among Estonian and Finnish physicians. Soz Praventivmed.

[17] Pärna K, Rahu K, Rahu M (2005). Smoking habits and attitudes towards smoking among Estonian physicians. Public Health.

[18] Paapsi K, Pärna K. Uuringu "Epidemioloogiline ja geneetiline tõendus tervishoiutöötajate suitsetamiskäitumise ja nikotiinisõltuvuse kohta" andmed kogutud. [Data collection of the project "Epidemiological and genetic evidence about smoking behaviour and nicotine dependnce among health professionals in Estonia" is completed]. Eesti Arst. 2015;94:697.

[19] Lohur L, Pärna K (2016). Arstide suitsetamine, sellealased hinnangud ja tähelepanu pööramine patsientide suitsetamisele. [smoking habits, smoking related opinions and attitudes towards patients' smoking habits among physicians in Estonia]. Eesti Arst.

[20] Ahmad OP, Boschi-Pinto C, Lopez AD, Murray CJL, Lozano R, Inoue M. Age standardization of rates: a new WHO standard. GPE Discussion Paper Series: No. 31. Geneva: World Health Organization; 2001.

[21] Cuzick JA (1985). Wilcoxon-type test for trend. Stat Med.

[22] Stata 12. StataCorp (2011). Stata statistical software: release 12.

[23] Doll R, Peto R, Wheatley K, Gray R, Sutherland I (1994). Mortality in relation to smoking: 40 years' observations on male British doctors. BMJ.

[24] Barengo NC, Sandström PH, Jormanainen VJ, Myllykangas MT (2004). Changes in smoking prevalence among Finnish physicians 1990–2001. Eur J Pub Health.

[25] Nelson DE, Giovino GA, Emont SL (1994). Trends in cigarette smoking among US physicians and nurses. JAMA.

[26] La Vecchia C, Scarpino V, Malvezzi I, Baldi G (2000). A survey of smoking among Italian doctors. J Epidemiol Community Health.

[27] Josseran L, King G, Guilbert P, Davis J, Brucker G (2005). Smoking by French general practitioners: behaviour, attitudes and practice. Eur J Pub Health.

[28] Leinsalu M, Grintšak M, Noorkõiv R (1999). Eesti Terviseuuring. Tabelid. Estonian health interview survey. Tables.

[29] Matsi A, Oja L. Eesti Terviseuuring 2006. Tabelid. Estonian Health Interview Survey 2006. Tables. Tallinn: Tervise Arengu Instituut; 2009.

[30] Taioli E, Wynder EL (1991). Effect of the age at which smoking begins on frequency of smoking in adulthood. N England J Med.

[31] Movsisyan NK, Varduhi P, Arusyak H, Diana P, Armen M, Frances SA (2012). Smoking behavior, attitudes, and cessation counseling among healthcare professionals in Armenia. BMC Public Health.

[32] Ohida T, Sakurai H, Mochizuki Y (2001). Smoking prevalence and attitudes toward smoking among Japanese physicians. JAMA.

[33] Zanetti F, Gambi A, Bergamaschi A (1998). Smoking habits, exposure to passive smoking and attitudes to a non-smoking policy among hospital staff. Public Health.

[34] Estonian Tobacco Act. Tallinn: Riigi Teataja; 2000. faolex.fao.org/docs/texts/est37797.doc. Accessed 18 Jan 2017.

[35] Estonian Tobacco Act. Tallinn: Riigi Teataja; 2005. https://www.riigiteataja.ee/en/eli/525032015017/consolide. Accessed 18 Jan 2017.

[36] Green Paper on Tobacco Policy. Tallinn: Ministry of Social Affairs; 2014. https://www.sm.ee/sites/default/files/content-editors/eesmargid_ja_tegevused/Tervis/Tervislik_eluviis/tubakapoliitika_roheline_raamat_18_12_13_12_en.pdf. Accessed 23 Jan 2017.

[37] Estonian Network of Health Promoting Hospitals and Health Services. http://www.ensh.org/members.php?id=48. Accessed 23 Jan 2017.

[38] Tervistedendavate haiglate ja terviseteenuste võrgustik. Tallinn: Terviseinfo; 2016. http://www.terviseinfo.ee/et/tervise-edendamine/tervishoiuasutuses/tervist-edendavate-haiglate-ja-terviseteenuste-vorgustik. Accessed 23 Jan 2017.

[39] Health promotion in hospitals. Tallinn: National Institute for Health Development; 2016. http://tai.ee/et/tegevused/tervise-edendamine/tervise-edendamine-tervishoiuasutustes. Accessed 25 Jan 2017.

